# Correction: Belfeki et al. State of the Art of Cardiac Amyloidosis. *Biomedicines* 2023, *11*, 1045

**DOI:** 10.3390/biomedicines14010033

**Published:** 2025-12-23

**Authors:** Nabil Belfeki, Nouha Ghriss, Mehran Monchi, Cyrus Moini

**Affiliations:** 1 Department of Internal Medicine and Clinical Immunology, Groupe Hospitalier Sud Ile de France, 77000 Melun, France; 2 Department of Intensive Care Medicine, Groupe Hospitalier Sud Ile de France, 77000 Melun, France; mehran.monchi@ghsif.fr; 3 Department of Cardiology, Groupe Hospitalier Sud Ile de France, 77000 Melun, France; cyrus.moini@ghsif.fr

**Error in Reference Citation**

In the original publication [1], reference 49 “Lanini, S.; Garbuglia, A.R.; Lapa, D.; Puro, V.; Navarra, A.; Pergola, C.; Ippolito, G.; Capobianchi, M.R. Epidemiology of HEV in the Mediterranean basin: 10-year prevalence in Italy. *BMJ Open* **2015**, *5*, e007110” has been mistakenly cited.

This reference has been deleted and the citation in the text has been replaced with the correct reference 21: “Gościniak, P.; Baron, T.; Milczarek, S.; Kostkiewicz, M.; Machaliński, B. Updates for the diagnosis and management of cardiac amyloidosis. *Adv. Clin. Exp. Med.* **2022**, *31*, 175–185”.

All the references from the 49th have been renumbered in the text and in the reference list.

The authors state that the scientific conclusions are unaffected. This correction was approved by the Academic Editor. The original publication has also been updated.
